# Does crowdfunding foster rural entrepreneurship?

**DOI:** 10.1007/s11187-026-01194-8

**Published:** 2026-03-11

**Authors:** Nicola Cortinovis

**Affiliations:** Utrecht University, Utrecht, The Netherlands

**Keywords:** Crowdfunding, Entrepreneurial finance, Social capital, US, Rural counties, R11, L26, G3

## Abstract

Entrepreneurs in rural areas face much greater difficulties than those located in cities, also with respect to the access to entrepreneurial finance. Recent developments in the provision of capital, however, have opened new opportunities for small firms and start-ups to obtain funding. In this empirical work, I hypothesize that crowdfunding provides crucial resources and support for rural-based entrepreneurs and that rural areas characterized by greater (bridging) social capital are better positioned to benefit from the opportunities of crowdfunding. Using a newly developed database linking crowdfunding campaigns to industry and counties in the U.S. (KIUS), county-level information on social capital and official U.S. census data, I test these hypotheses. My findings indicate that crowdfunding is indeed positively related to the number of ventures operating in the industry-location in the following period. In addition, this relationship is stronger for counties with higher levels of bridging social capital and of civic engagement. The results are robust to a number of checks, including a placebo test and matching exercises.

## Abstract (plain language summary)

Access to capital through crowdfunding is critical for rural counties: obtaining funds through Kickstarter is associated with an increase in the number of business ventures operating in rural areas. Rural-based entrepreneurs have to face a number of challenges to establish their ventures. Among these challenges, a crucial one pertains access to capital, which is much less available and difficult to obtain outside the city. The use of crowdfunding however may represent an important alternative to traditional funding channels, like banks and venture capitalists. In this paper, I show that providing resources to rural entrepreneurs through crowdfunding is associated to a higher number of ventures operating in the industry in the location which received the funds. Interestingly, the effect of crowdfunding is strongest for those areas with higher rates of volunteering and higher connections between people with different socio-economic backgrounds. These results suggest to practitioners and policy-makers that, one the one hand, rural entrepreneurs can successfully exploit crowdfunding for their ventures; on the other hand, social connections are essential for taking advantage of opportunities offered by crowdfunding.

## Introduction

From an economic geography and entrepreneurship perspective, start-ups and small firms in rural areas face much greater difficulties than their urban counterparts do (Glaeser et al., 1992; Henderson et al., 1995; Huiban, 2011). At the same time, a growing strand of literature has highlighted how entrepreneurship and innovation do take place also outside the city (Aguilar, 2021; Cuéllar-Fernández et al., 2024; Shearmur, 2012). While some factors typical of rural areas (e.g., natural resources, low cost of living) have been used to explain rural entrepreneurship (Fanjul et al., 2023; Korsgaard et al., 2015), other factors—particularly technological change—may also play a role. For instance, the emergence of digital technologies has spurred path-breaking innovations and the entry of new players in entrepreneurial finance (Block et al., 2018). One of these changes is the emergence of crowdfunding, a novel way to gather capital resources for ventures and projects that may facilitate and support rural entrepreneurship dynamics (Dunkley, 2016; Mollick & Kuppuswamy, 2016; Mollick & Robb, 2016; Schwartz, 2012). While theoretically motivated, the real impact of crowdfunding in rural areas has not received much empirical support, except for some indications that firms located outside urban areas rely on online platforms for funding to overcome geographical barriers (Bernardino et al., 2016; Cumming et al., 2021).

This article aims to fill this gap and tries to better understand whether and under what conditions crowdfunding may represent an important driver of rural entrepreneurship in the U.S. Theoretically, this paper builds on the concept of the entrepreneurial ecosystem (EE) as a construct to identify crucial factors in the local context that explain productive entrepreneurship (Schrijvers et al., 2024; Stam & Van De Ven, 2021). Adapting the EE lenses to the context of rural entrepreneurship (Aguilar, 2021; Mayer et al., 2016; Muñoz & Kimmitt, 2019a), this paper highlights how access to finance through crowdfunding can supplement some of the structural weaknesses that typically characterize rural regions (e.g., lack of financial resources, limited chances for knowledge spillovers and inputs from experts or other entrepreneurs) (Agrawal et al., 2015; Colombo et al., 2015; Martínez-Climent et al., 2021) while leveraging other local characteristics (high levels of social capital) (Frimanslund et al., 2023; Korsgaard et al., 2015). Focusing on the context of US counties, I empirically test whether crowdfunding is associated with a greater number of firms active in industries locally and how different dimensions of social capital affect this relation. To this end, I analyze recently developed and highly granular data linking Kickstarter projects to industries (KIUS) (Mitra et al., 2024) and leverage different measures of social capital in the US (Chetty et al., 2022; Rupasingha et al., 2006). My empirical analysis reveals a positive relationship between successful crowdfunding campaigns and the number of firms operating in the industry and location in the following year, suggesting that crowdfunding may stimulate entrepreneurship in rural counties. Interestingly, my findings indicate that the relationship is concentrated in areas characterized by high bridging social capital (economic connectedness, volunteering rate, bridging-type of organizations and presence of NGOs) and low bonding social capital (clustering). The findings are robust to numerous checks, in terms of definitions of rural areas, placebo tests and matching exercises.

My work offers some relevant contributions to the literature on rural entrepreneurship and entrepreneurial ecosystems. First, I investigate and provide evidence of the crucial role of finance—and innovations in the finance industry—in supporting local entrepreneurship in rural areas. I suggest that crowdfunding is a particularly important innovation, as it both alleviates the credit constraints that entrepreneurs in peripheral regions face and potentially offers a platform for fine-tuning ideas and obtaining knowledge spillovers. Second, I contribute to the emerging literature highlighting the potential for innovation and entrepreneurship in rural areas. Specifically, I show that one of the features typically associated with rural areas (high social capital) is synergic to crowdfunding, as suggested by earlier literature (Giudici et al., 2018). Finally, my analysis is the first to exploit a large-scale database linking crowdfunding to industries and locations in the U.S., which offers a new way to empirically study crowdfunding and entrepreneurial dynamics.

The paper is structured as follows. The first section provides an overview of the main theoretical aspects of my work and connects to the literature on EE, crowdfunding and rural entrepreneurship. In the third section, I present the data and methods I used before moving to the empirical results (in the fourth section) and conclusions.

## Theoretical framework

### Entrepreneurial ecosystems in rural areas

The concept of entrepreneurial ecosystems (EE) has become crucial for understanding the drivers and challenges that small firms and entrepreneurs face and how these drivers and challenges are shaped by the local context (Audretsch & Belitski, 2017; Spigel & Harrison, 2018; Stam & Van De Ven, 2021). The appeal and power of EE as a construct comes from its ability to systematically analyze the factors and conditions behind (productive) entrepreneurship outcomes. While the main dimensions of EE – institutional arrangements, resource endowments and outcomes—are quite generally defined (Schrijvers et al., 2024; Stam & Van De Ven, 2021), the more detailed components of EE are more or less explicitly connected to spatial agglomeration and urban environments. From high accessibility and quality of infrastructure to thick labor markets and high levels of human capital, most of the defining features of successful EE are inherently urban. It is thus not surprising that much of the empirical literature that uses EE also focuses on cities (Audretsch & Belitski, 2017; Spigel, 2017). In the context of rural entrepreneurship, the underlying urban bias in EE has been recently subjected to discussion, with scholars questioning the extent to which EE as a concept is actually applicable to non-urban areas (Aguilar, 2021; Mayer & Motoyama, 2020; Muñoz & Kimmitt, 2019a).

The differences between urban and rural settings and the more challenging conditions for entrepreneurs in rural areas have long been recognized in the literature (Acs & Kallas, 2008). Together, the structural weaknesses, negative perceptions and obstacles faced by rural entrepreneurs (Aguilar, 2021; del Olmo-García et al., 2023; Figueroa-Armijos & Berns, 2022; Figueroa-Armijos et al., 2012; Muñoz & Kimmitt, 2019a) have often been interpreted as evidence of underdeveloped EEs. Other research efforts, however, have highlighted how, rather than being absent or weaker, rural EEs are simply different from urban EEs (Aguilar, 2021; Almeida & Daniel, 2025; Mayer & Motoyama, 2020; Naldi et al., 2021).

In particular, rural EEs rely less on size, infrastructure and technology while they exploit alternative configurations of factors, especially those related to place-specific characteristics and external connections (Mayer et al., 2016; Meili & Shearmur, 2019). In terms of endogenous resources, rural entrepreneurs are able to leverage specific strengths of their location, such as dense social capital (Korsgaard et al., 2015; Muñoz & Kimmitt, 2019a) and local amenities (Naldi et al., 2021). The embeddedness of entrepreneurial practices and processes in local culture and geography in turn provides important advantages to rural entrepreneurs. Through the mobilization of locally available assets and networks (Anderson, 2000; Garrod et al., 2006; Müller & Korsgaard, 2018), ventures based in rural areas can achieve growth and capture important market niches (Alsos et al., 2014; Baù et al., 2019; Wojan & Nichols, 2018). Moreover, cultivating and taking advantage of external relationships to develop a competitive advantage (often linked to local cultural and natural assets) is also a prominent element of rural entrepreneurship (Mayer & Motoyama, 2020; Mayer et al., 2016). External connections allow to complement local characteristics (e.g., access to knowledge or specific competences, augmented social capital) and to access larger markets and pools of consumers (Korsgaard et al., 2015; Mayer et al., 2016; Moyes et al., 2015). Interestingly, while the importance of relations with parties outside the local *milieu* has long been seen as an important factor (Basu et al., 2025; Smallbone et al., 2003; Virkkala, 2007), recent contributions suggest that ICT and the internet facilitate the development and exploitation of external connections (Deller et al., 2022; Nambisan et al., 2019; Smith et al., 2017), including for rural entrepreneurs (Conroy & Low, 2022; Romero-Castro et al., 2023; Yaşlak et al., 2023).

Owing to these recent contributions to the rural entrepreneurship literature, it is now better understood how EEs leverage local factors in different ways, leading to different configurations in different places without detriment to their success (Audretsch & Belitski, 2021; Muñoz & Kimmitt, 2019a; Schrijvers et al., 2024; Spigel, 2017).

### Entrepreneurship in rural areas: what role does crowdfunding play?

Nonetheless, one of the most important challenges faced by ventures in rural EEs is accessing entrepreneurial finance (Figueroa-Armijos & Berns, 2022; Muñoz & Kimmitt, 2019a; Stam & Van De Ven, 2021). While access to capital is one of the pillars within the EE framework, its role and importance have been understudied (Frimanslund, 2022; Frimanslund et al., 2023), especially for rural entrepreneurs (del Olmo-García et al., 2023; Figueroa-Armijos & Berns, 2022). This is to some extent surprising given the significant changes that have occurred in the field and the entry of new players in entrepreneurial finance over recent decades (Block et al., 2018). Innovation in digital technologies and finance has led to the emergence of crowdfunding as a possible new tool for entrepreneurs to access capital resources (Block et al., 2018; Mollick, 2014).

These recently emerged online platforms for crowdfunding have translated into new opportunities for entrepreneurs and creators to seek and obtain funds (Dunkley, 2016; Figueroa-Armijos & Berns, 2022; Mollick, 2014). These developments have been especially important for groups, sectors and locations, such as rural entrepreneurs (Bernardino et al., 2016; Breznitz & Noonan, 2020; Cumming et al., 2021), for which traditional financial channels are usually less open or available (Lee & Brown, 2017; Mollick & Robb, 2016; Sorenson et al., 2016). Moreover, different contributions highlight how crowdfunding provides more than financial resources, conveying information about markets (Kim & Viswanathan, 2019; Paschen, 2017) and new ideas (Agrawal et al., 2014; Martínez-Climent et al., 2021) as well as helping entrepreneurs expand social capital (Cai et al., 2021; Colombo et al., 2015; Smith et al., 2017). From this perspective, crowdfunding represents a potentially crucial tool for start-ups and small firms in non-urban areas, since it supports and complements the endogenous factors and external relations upon which rural EEs typically rely (Korsgaard et al., 2015; Mayer & Motoyama, 2020; Müller & and Korsgaard, 2018; Yaşlak et al., 2023).

#### Access to finance and knowledge spillovers through crowdfunding

Scholars working on crowdfunding and rural entrepreneurship have realized how crowdfunding may offer a solution to some of the challenges faced by start-up and small firms in rural areas (Bernardino et al., 2016; Cumming et al., 2021; Sorenson et al., 2016).

In a context where rural entrepreneurs face greater difficulties in accessing capital for their ventures (Lee & Brown, 2017; Ughetto et al., 2019), crowdfunding represents a financial tool which can democratize access to capital (Mollick & Robb, 2016; Sorenson et al., 2016) and help mitigate the liability of distance and financial barriers faced by rural EEs (Figueroa-Armijos et al., 2012). In this sense, by making it possible to connect and pitch a project to a much wider audience than few locally available financial institutions do (Grilli, 2019; Mollick & Robb, 2016; Paschen, 2017), crowdfunding platforms effectively create new funding opportunities to be exploited for rural-based firms (Belleflamme et al., 2014; Eldridge et al., 2021). For example, in the context of equity-based crowdfunding in the UK, research has shown that firms located outside metropolitan areas are more likely to have higher chances of obtaining resources through crowdfunding (Cumming et al., 2021). Similarly, Portuguese firms in rural areas are found to rely more heavily on crowdfunding for gathering funds than their urban counterparts are (Bernardino et al., 2016). In addition, by using narratives that resonate with specific communities of interest (Figueroa-Armijos & Berns, 2022; Muñoz & Kimmitt, 2019b), crowdfunding enhances the visibility and legitimacy of rural ventures. These, in turn, can reduce issues of information asymmetries, monitoring and informal networks, which are often used to explain the uneven geography of entrepreneurial finance (Cowling et al., 2021; van Rijnsoever, 2020). The successful acquisition of resources through crowdfunding has also been shown to contribute to placing locations on the map of venture capitalists (Sorenson et al., 2016) and business angels (Yu et al., 2017), potentially facilitating access to financing at later stages.

A second element identified in the literature is the ability of crowdfunding platforms to provide knowledge spillovers, insights and ideas through interactions between entrepreneurs and backers (Agrawal et al., 2014; Elia et al., 2020; Martínez-Climent et al., 2021; Paschen, 2017). From the point of view of the knowledge spillover theory of entrepreneurship (Acs et al., 2013), urban-based entrepreneurs are able to benefit from agglomeration economies (e.g., access to technical and specialized knowledge and a variety of knowledge inputs) to generate new ideas and pursue their projects (Duranton & Puga, 2001; Henderson et al., 1995; Huiban, 2011). In contrast, entrepreneurs based in rural areas are largely deprived of these benefits. By establishing a communication channel with possible consumers and experts (Kim & Viswanathan, 2019; Mollick & Nanda, 2016), crowdfunding effectively allows (rural) entrepreneurs to test the market potential of their project, tap into the knowledge of a larger crowd and fine-tune their own idea (Elia et al., 2020; Martínez-Climent et al., 2021; Mollick & Kuppuswamy, 2016). For example, Elia et al. (2020) discuss how Kickstarter facilitates entrepreneurial processes not only through the transfer of capital resources but also through inspiration, networking and provision of advice and insights. Similarly, Eiteneyer et al. (2019) show that the mobilization of entrepreneurs’ social capital through crowdfunding is associated with knowledge sharing and codevelopment, which ultimately affect the innovativeness of the product offering. Overall, empirical and anecdotal evidence suggests that these knowledge inflows and spillovers are rather valuable for creators and entrepreneurs (Agrawal et al., 2014; Martínez-Climent et al., 2021), especially when fostering connections outside the specific rural context of the entrepreneur (Colombo et al., 2015; Korsgaard et al., 2015).

Against this backdrop, the emergence of crowdfunding platforms can be seen as a possible tool to facilitate rural entrepreneurship. Firstly, the provision of financial resources through crowdfunding may fill an important gap in access to entrepreneurial funding and in establishing greater legitimacy and visibility for entrepreneurs in rural areas (Block et al., 2018; Figueroa-Armijos & Berns, 2022; Figueroa-Armijos et al., 2012; Sorenson et al., 2016). Secondly, strategic network connections are critical for obtaining advice and insights in the context of rural entrepreneurship (Eiteneyer et al., 2019; Korsgaard et al., 2015; Paschen, 2017). As online crowdfunding platforms may offer an alternative for obtaining such inputs (Agrawal et al., 2014; Martínez-Climent et al., 2021), I theorize that crowdfunding plays an important role in fostering entrepreneurial activity in rural areas.

More specifically:

- *H1: The provision of resources through crowdfunding is positively associated with the number of establishments*^1^*in the targeted industry and location.*

#### Social capital and crowdfunding in rural areas

One of the most important facets defining rural EEs is the dense network of social capital relations (Korsgaard et al., 2015; Ring et al., 2010). This defining feature, often highlighted as one of the important advantages that rural entrepreneurs can rely on (Baù et al., 2019; Lang & Fink, 2019; Müller & and Korsgaard, 2018), is synergistic with the mechanisms through which crowdfunding operates. Two well-known factors highlighted in the crowdfunding literature are that i) family and friends play an important role in financially supporting projects, especially in early stages, and that ii) the probability of obtaining funds drastically increases once a project is more than 50% funded (Agrawal et al., 2015; Colombo et al., 2015; Kuppuswamy & Bayus, 2015, 2018). With various contributions underlining the role of social capital in successfully leveraging crowdfunding platforms (Butticè et al., 2017; Cai et al., 2021; Camilleri & Bresciani, 2022), it can be argued that crowdfunding platforms may thus help rural entrepreneurs mobilize their local relationships (which the crowdfunding literature refers to as “external social capital” (Cai et al., 2021; Colombo et al., 2015)) to pass the critical threshold and obtain funding from backers outside their local networks. Exploiting this synergy between social capital and crowdfunding (Giudici et al., 2018), entrepreneurs in rural areas characterized by higher levels of social capital may be better positioned to orchestrate their network and obtain funding and insights for their projects.

Moreover, social capital can take different configurations. The literature typically distinguishes between bonding and bridging social capital. Bonding social capital refers to dense social structures characterized by strong ties among closely connected individuals, such as family members, or tightly knit local networks (Banfield, 1958; Deller et al., 2018; Putnam, 2001). In rural entrepreneurial contexts, these strong ties can provide trust and support, which is particularly relevant in the case of crowdfunding (Agrawal et al., 2015). However, high levels of bonding social capital are also associated with conformity bias and social pressure, reducing the scope for innovation and the emergence of new activities (de Vaan et al., 2019; North, 2005). In contrast, bridging social capital consists of more inclusive, outward-looking networks that connect individuals with diverse socioeconomic backgrounds (Cai et al., 2021; Deller et al., 2018; Putnam, 2001). These cross-cutting connections facilitate access to non-redundant knowledge, external resources, and broader markets (Cortinovis et al., 2017), enabling entrepreneurs to identify opportunities, access critical insights and fine-tune their ideas (Butticè et al., 2017; Deller et al., 2018; Giudici et al., 2018).

Building on this idea and following Giudici et al. (2018), I anticipate a potential synergistic effect between rural EEs and crowdfunding. In particular, I expect that the impact of crowdfunding on the number of establishments within an industry location will be stronger in rural regions exhibiting higher overall levels of social capital. While my hypothesis does not distinguish between bonding and bridging ties, I use the data to examine whether these different forms of social capital have distinct moderating impacts.

Specifically, I hypothesize the following:

- *H2: The relationship between crowdfunding and the number of establishments is stronger for areas characterized by high levels of social capital.*

## Data and methodology

### Data sources

To test these hypotheses, my analysis focuses on the use of Kickstarter—one of the main platforms for reward-based crowdfunding—in rural counties in the United States. The two main sources of data for my analysis are the County Business Patterns (CBP) database (Eckert et al., 2020) and the recently developed KIUS database (Kickstarter and Industries in the U.S.) (Mitra et al., 2024). The CBP database provides detailed information on the industry composition of US counties over the years. While the official data include numerous missing values to protect privacy—an issue particularly relevant for rural areas—Eckert and coauthors developed an algorithm to impute and fill in the missing values, thus providing relevant coverage, although only for the years up to 2016 (Eckert et al., 2020)^2^. The KIUS database instead leverages web-scraped data on Kickstarter and Large Language Models to classify approximately 300,000 crowdfunding projects into the most likely NAICS code, thus allowing us to place crowdfunding in a specific industry, location and year. The appendix contains more information about the validation steps carried out to establish how the consistency and reliability of the database developed. In brief, the KIUS data have been developed with the support of AI (ChatGPT API) to link specific Kickstarter projects to the relevant industry. The data developed through this classification task carried out by AI have been validated by humans (one expert and five students) and achieved a reassuring level of agreement (54% agreement; *Kohen’s k*: 0.47; *Gwet AC1*: 0.53). In addition to these data sources, I include other information from the US Census Bureau (e.g., GDP by county, population in rural areas), from the Federal Deposit Insurance Corporation (i.e., total deposits in bank branches by county^3^) and from extant studies on social capital (Chetty et al., 2022; Rupasingha et al., 2006).

One of the empirical challenges in studying rural entrepreneurship is correctly identifying which areas can be classified as rural. To account for the multifaceted nature of rural areas (Cattivelli, 2024; Ratcliffe et al., 2016), I define rural areas as characterized by four features: 1) lack of large urban centers; 2) majority of the population living in rural areas; 3) low population density; and 4) geographical remoteness from large urban centers. Empirically, these four features are mapped in four necessary conditions to identify rural counties: 1) having fewer than 10,000 living in urban areas; 2) having at least 80% of the population living in rural areas; 3) having a population density < 250 people per square mile (< 95 people per square km); and 4) having a distance of more than 50 miles (80 km) from the county border to the closest urban core (city of 50,000 people or more). These criteria are well aligned with the empirical literature, and they combine insights from different contributions in the field (Cattivelli, 2024; Isserman et al., 2009; Kärnä & Stephan, 2022; Ratcliffe et al., 2016). For example, Figueroa-Armijos and Johnson (2013) define rural regions on the basis of small urban populations and low population density but do not include an element of distance from larger urban cores. Cuéllar-Fernández et al. (2024) instead use similar criteria as those applied here, although with less stringent thresholds (e.g., population density < 300 people per square km), and they also consider a demographic aspect (i.e., higher average age).^4^

Overall, my analysis covers the period 2009–2016, with the first year being chosen as the first year of operation of Kickstarter and the last year being defined by the data availability of CBP (Eckert et al., 2020). Combining and aggregating the abovementioned data sources allows me to study the relationship of interest at a rather fine-grained level, using data disaggregated by 3-digit NAICS code, county and year.

### Modeling

To test my hypotheses on the relationship between crowdfunding and the number of ventures in rural areas, I estimate the following regression model:

$${number\_plants}_{i,c,t}={\beta *CF}_{i,c,t-1}+\gamma {\boldsymbol{*}{\boldsymbol{C}}{\boldsymbol{o}}{\boldsymbol{n}}{\boldsymbol{t}}{\boldsymbol{r}}{\boldsymbol{o}}{\boldsymbol{l}}{\boldsymbol{s}}}_{i,c,t-1}^{1}+\theta *{{\boldsymbol{C}}{\boldsymbol{o}}{\boldsymbol{n}}{\boldsymbol{t}}{\boldsymbol{r}}{\boldsymbol{o}}{\boldsymbol{l}}{\boldsymbol{s}}}_{c,t-1}^{2}+{\vartheta }_{i,c}+{\tau }_{t}+{\varepsilon }_{c}$$

where the variable $number\_plants$ measures the number of establishments^5^ operating in the specific industry *i*, in county *c*, in year *t*. As the dependent variable is expressed as integers (number of establishments), the regression will be estimated as a fixed effect Poisson model (Bergé, 2018). The main regressor of interest is $CF$, which captures the exposure of the industry location to crowdfunding in the previous year. Following the literature (Breznitz & Noonan, 2020; Mollick & Kuppuswamy, 2016), I use different measures of exposure to crowdfunding, specifically: i) whether at least one project was funded in the specific industry-location; ii) whether at least one large (> 5000 USD) project was funded in the specific industry-location; and iii) the total amount raised in the specific industry-location. As controls, I include one variable, relatedness density, which varies by industry-location-year and captures how close the focal industry *i* is to the current portfolio of specializations of the county (Cortinovis et al., 2017; Hidalgo et al., 2007) (see Appendix 2 for details on the construction of the variable). I argue that this is an important factor in accounting for local sectoral structure (Audretsch & Belitski, 2021). I add as further control variables the level of GDP of the county to hold constant the overall state of the local economy, the contribution of financial industries to local GDP to control for access to finance, the number of patents to control for innovation dynamics and the number of employees in the county to control for possible agglomeration effects. Conceptually, these variables are important determinants of EE (Stam & Van De Ven, 2021). Importantly, my regression model includes both industry-location and year fixed effects. As I use within industry-location variation to identify the parameters, the fixed effects absorb time constant (or very slowly varying) factors (e.g. culture, infrastructure).

My second hypothesis focuses on the role of social capital as a possible mediator in the relationship between crowdfunding and establishments. Given the difficulty of finding reliable measures of social capital and the inclusion of fixed effects, I rely on county-level information and split my sample of rural regions into those in the top and bottom quartiles in terms of social capital. Essentially, I estimate the same model for different subsamples defined on the level of each rural county social capital level in 2020. With respect to the data, Chetty et al. (2022) provide information on three dimensions of social capital, namely, civic participation (volunteering rate, linked to bridging social capital (Putnam, 2001)), economic connectedness (connection between people of different socio-economic conditions, also linked to bridging social capital Chetty et al., 2022; Putnam, 2001)) and social cohesiveness (how frequently friends have mutual friends, more connected to bonding social capital (Chetty et al., 2022)). In my analysis, I consider all three dimensions. Given the time mismatch between my period of analysis and the data from the Social Capital Atlas, I further test the role of social capital using information from 2009 from Rupashinga et al. (2006)^6^. This database uses more traditional measures of social capital, which are based on membership in or presence in the county of different types of organizations (e.g., political parties, religious associations, bowling and sport clubs). From the data developed by Rupashinga et al. (2006), I consider the dimensions of social capital that align best with those of Chetty et al. (2022), namely, a measure of general social capital (bridging and bonding), a measure of bridging social capital^7^ and a measure of civic engagement proxied by the number of nonprofit organizations (excluding those with an international scope).

### Descriptive statistics

**Fig. 1:**
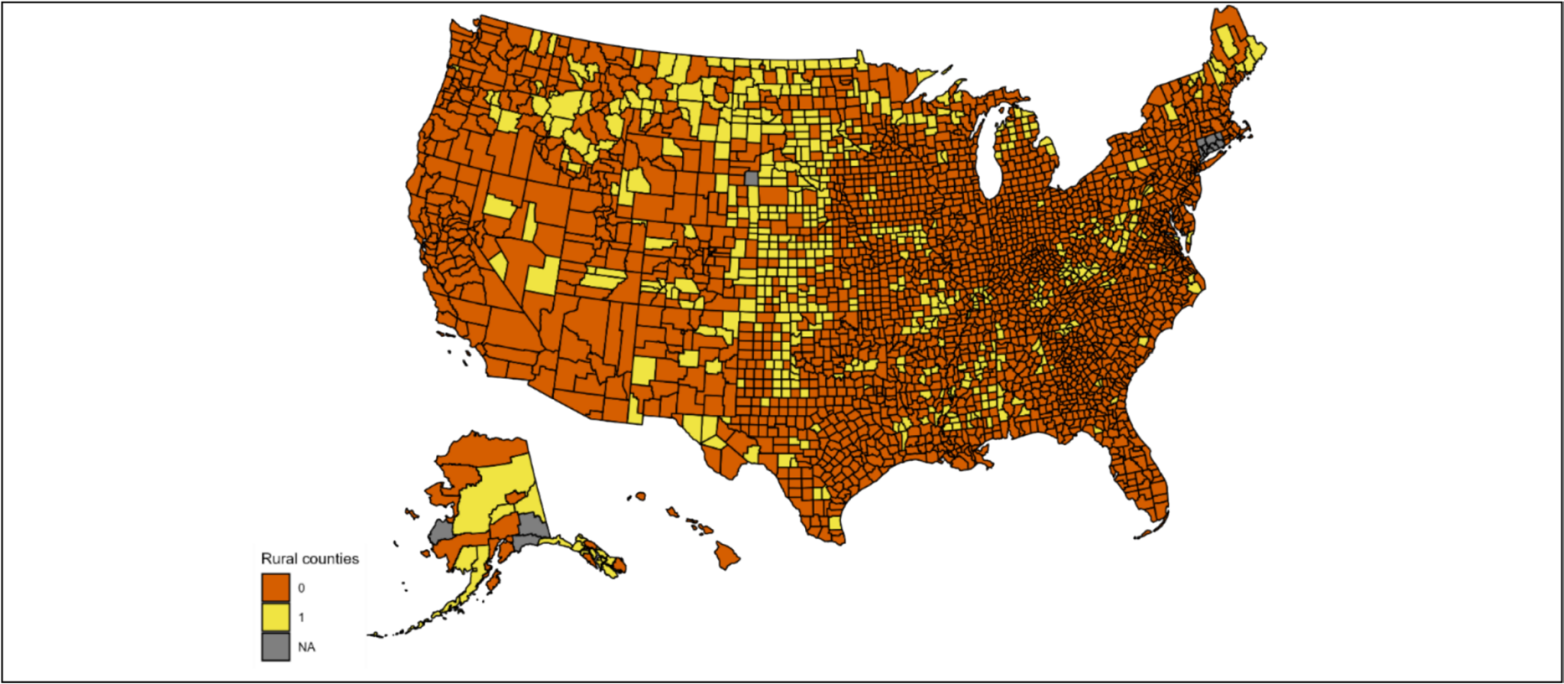
Rural and non-rural counties

The empirical analysis below focuses on the impact of crowdfunding in rural US counties. Using information from the Census Bureau in 2010, I categorize counties into rural or non-rural based the four different factors discussed in the previous section. Figure 1 shows the spatial distribution of counties categorized as rural (in yellow). Among the 3142 counties in the U.S., approximately 18% (565) are considered rural. The map also clearly shows how rural counties (yellow) are located predominantly in the central and western parts of the U.S., including much of the Midwest and Mountain West regions. Rural counties are also present in the eastern states (Maine, Vermont, West Virginia), although with lower concentrations.

In Table 1, I compare the median and mean of counties in the rural categories against the others over a number of dimensions. As expected, rural counties report relatively lower values than urban counties do for all the variables. For instance, the median income in rural areas is between 11 and 15% lower than that in their urban counterparts. Interestingly, while measured rather recently, the difference in broadband coverage between rural and nonrural areas does not appear to be particularly high^8^.

**Table 1:** Comparison between rural and non-rural counties

	Rural counties (*n* = 565)		Non-rural counties (*n* = 2631)	
	Median	Mean	Median	Mean
Population (2010)	5710	9175	33704	115,403
Median income (2010)	38,709	38,866	43,240	45,315
Private non-farm est. (2009)	138	207.8	726	2778
% Households with broadband (2019)	72.4	71.16	77.3	76.21

The descriptive statistics in Tables 2 and 3 below provide a snapshot of the data used in the analysis. In particular, the descriptive statistics reveal how crowdfunding is rather concentrated overall but potentially allows for the gathering of a substantial amount of resources, as the maximum value of crowdfunded resources in rural areas is approximately 200,000 USD. For reference, inspecting the data indicates that, conditional on having at least one successful campaign, the average amount of capital raised is 11,000 USD. The correlations in Table 3 show, on the one hand, that the number of establishments operating in an industry is positively but weakly related to crowdfunding and, on the other hand, that the more related an industry is to the industries already presented in the area, the lower the probability of it relying on crowdfunding (negative correlation between relatedness density and crowdfunding).

**Table 2:** Descriptive statistics

Variable	Definition	N	Mean	St. Dev	Min	Max
# Establishments	Count of employment-generating plants	178,905	5.059	8.365	1	200
CF (dummy)	Presence of at least 1 CF projects	178,905	0.001	0.032	0	1
Large CF (dummy)	Presence of at least 1 large (> 5000 USD) CF projects	178,905	0.0005	0.022	0	1
Amount raised (000 s)	Amount raised through CF	178,905	0.012	0.806	0.000	197.693
CF projects	Number of CF projects	178,905	0.001	0.047	0	8
Relatedness density	Strength of links between local specializations and focal industry	178,905	8.615	8.268	0.000	86.060
Tot. employment	Employment level in the county	178,905	2,446.867	2,812.247	0	21,181
Tot. patent count	Number of patents developed in the county	178,905	2.523	12.785	0	257
GDP	Gross Domestic Product of the county	178,201	343,848.400	379,808.600	4,040	4,884,983
Finance	Portion of GDP of the financial sector	178,905	63,481.370	66,212.430	0	527,151
Bank deposits	Deposits held in local banks	178,905	169,846.200	185,176.600	0	1,745,775

**Table 3:** Correlation table

		**1**	**2**	**3**	**4**	**5**	**6**	**7**	**8**	**9**	**10**	**11**
Establishments	**1**	1										
CF (dummy)	**2**	.03	1									
Large CF (dummy)	**3**	.02	.71	1								
Amount raised (000 s)	**4**	.02	.46	.58	1							
CF projects	**5**	.02	.83	.63	.42	1						
Relatedness density	**6**	.00	-.01	-.01	-.00	-.01	1					
Tot. employment	**7**	.39	.05	.04	.02	.04	-.23	1				
Tot. patent count	**8**	.15	.04	.04	.02	.03	-.06	.38	1			
GDP	**9**	.29	.03	.03	.02	.03	-.13	.73	.25	1		
Finance	**10**	.40	.05	.04	.03	.05	-.20	.88	.37	.67	1	
Bank deposits	**11**	.32	.04	.03	.02	.03	-.16	.76	.22	.60	.74	1

## Results

To empirically test my two hypotheses, I estimate the model presented in the last section via a fixed effects Poisson regression. The results of the baseline estimations, in which different proxies for exposure to crowdfunding are used, are reported in Table 4. The first column of Table 4 reports the results including only the control variables, which present overall the expected signs. From the second to the last columns of Table 4, different measures of crowdfunding are used. Nonetheless, the coefficients consistently indicate a positive relation (although only significant at the 10% level in the second column) between obtaining funding through Kickstarter and the number of establishments operating in the same industry the following year. In my preferred specification, which uses *Large CF (dummy)* as an explanatory variable, crowdfunding increases the number of establishments in the receiving industry location the following year by roughly 3% (exp(.033) = 1.033). This provides evidence confirming what I theorized in Hypothesis 1, namely, that crowdfunding is positively associated with the number of establishments operating in the same industry the following year.

**Table 4:** Baseline regressions

	CV only	CF	Large CF	Amount	Projects
CF (dummy)		0.020 +			
		(0.010)			
Large CF (dummy)			0.033*		
			(0.014)		
Amount raised (000 s)				0.001*	
				(0.000)	
CF projects					0.008
					(0.006)
Relatedness density	0.000	0.000	0.000	0.000	0.000
	(0.000)	(0.000)	(0.000)	(0.000)	(0.000)
Tot. employment (log)	0.147***	0.147***	0.147***	0.147***	0.147***
	(0.033)	(0.033)	(0.033)	(0.033)	(0.033)
Tot. patent count	0.000	0.000	0.000	0.000	0.000
	(0.000)	(0.000)	(0.000)	(0.000)	(0.000)
GDP (log)	0.055***	0.055***	0.055***	0.055***	0.055***
	(0.014)	(0.014)	(0.014)	(0.014)	(0.014)
Finance (log + 1)	−0.001	−0.001	−0.001	−0.001	−0.001
	(0.003)	(0.003)	(0.003)	(0.003)	(0.003)
Bank deposits (log + 1)	0.003**	0.003**	0.003**	0.003**	0.003**
	(0.001)	(0.001)	(0.001)	(0.001)	(0.001)
Num.Obs	151,257	151,257	151,257	151,257	151,257
Pseudo R2	0.645	0.645	0.645	0.645	0.645
NAICS3d-County FE	X	X	X	X	X
Year FE	X	X	X	X	X
Clustered SE	County	County	County	County	County

+ *p* < 0.1, * *p* < 0.05, ** *p* < 0.01, *** *p* < 0.001

To test my second hypothesis, I leverage two sources of information on social capital: Chetty et al. (2022) and Rupashinga (2006). These databases provide information on a number of variables. Specifically, for the Social Capital Atlas (Chetty et al. (2022)), I used three variables to capture different types of social capital: i) civic engagement, proxied by the share of people volunteering in organizations and conceptually connected to local altruism (Giudici et al., 2018; Putnam, 2001); ii) economic connectedness, capturing the share of high socio-economic status individuals who are friends of individuals of low socio-economic status (Putnam, 2001); and iii) social cohesiveness, capturing the cliques of mutual friends (Chetty et al., 2022). From the database by Rupashinga et al. (2006), I focus on similar variables, namely, i) a measure of general social capital based on participation in different organizations (e.g., sports, bowling, trade unions, political parties) (Knack & Keefer, 1997); ii) a measure of bridging social capital proxied by participation in civic organizations, sport and fitness clubs and bowling associations but excluding religious organizations (Deller et al., 2018; Putnam, 2001); and iii) a measure of civic engagement proxied by the number of non-profit organizations (excluding those with an international scope) (Putnam, 2001; Rupasingha et al., 2006).

To investigate the role of social capital, I subset my sample of rural regions into two groups on the basis of areas in the top and bottom quartiles of each of the six social capital variables (Cortinovis et al., 2017). Table 5 below reports my findings. For the sake of brevity, I focus only one proxy of exposure to crowdfunding (*Large CF (dummy)*), which, in line with the literature, I consider my preferred proxy (Mollick & Kuppuswamy, 2016; Mollick & Robb, 2016). The control variables were included but, for brevity, are not reported in Table 5.

**Table 5:** The impact of social capital

Social Capital by Chetty et al. (2022)						
	Low Altruism	High Altruism	Low Econ. Conn	High Econ. Conn	Low Clustering	High Clustering
Large CF (dummy)	−0.413***	0.042***	−0.122	0.024**	0.026*	−0.107
	(0.010)	(0.008)	(0.174)	(0.009)	(0.011)	(0.148)
Num.Obs	35,606	36,895	35,578	33,163	36,574	36,898
Pseudo R2	0.563	0.661	0.630	0.558	0.683	0.559
Control vars. included	X	X	X	X	X	X
NAICS3d-County FE	X	X	X	X	X	X
Year FE	X	X	X	X	X	X
Clustered SE	County	County	County	County	County	County
Social Capital by Rupashinga et al. (2006)						
	Low SK	High SK	Low Brid. SK	High Brid. SK	Low Non-profit org	High Non-profit org
Large CF (dummy)	0.107	−0.002	−0.147	0.027***	−0.223	0.042***
	(0.114)	(0.028)	(0.157)	(0.004)	(0.147)	(0.009)
Num.Obs	37,571	32,106	44,919	37,897	24,507	52,473
Pseudo R2	0.611	0.445	0.378	0.687	0.254	0.735
Control vars. included	X	X	X	X	X	X
NAICS3d-County FE	X	X	X	X	X	X
Year FE	X	X	X	X	X	X
Clustered SE	County	County	County	County	County	County

+ *p* < 0.1, * *p* < 0.05, ** *p* < 0.01, *** *p* < 0.001

When segmenting the sample along different social capital dimensions, I obtain further interesting results. Considering the more recent measures of social capital, counties characterized by greater participation in volunteering activities, which Chetty et al. link to civic engagement and which I connect to local altruism (Giudici et al., 2018), report a stronger and significant coefficient (second column in the top part of Table 5) than areas in the lower part of the distribution in terms of volunteering, for which a negative and significant relation is found (first column in the top part of Table 5). This suggests that crowdfunding platforms are indeed more effective in areas characterized by high civic engagement, which is in line with previous findings in the literature. I find comparable results when considering economic connectedness (third and fourth columns in the top part of Table 5). Interestingly, in the case of clustering, I find opposite results, with a positive and significant coefficient found only for counties with clustering scores lower than the median (last two columns in the top part of Table 5). The results from the subsamples based on Rupashinga et al. (2006) confirm and complement the previous findings. Interestingly, when considering social capital as a combination of bridging and bonding relations, no significant result is produced (first two columns in the bottom part of Table 5). While surprising, this finding is in line with the idea that social capital may actually deter entrepreneurship and innovation (de Vaan et al., 2019; Deller et al., 2018). However, when focusing on bridging social capital alone, the model confirms the previous results: the impact of crowdfunding is positive and significant only in the subsample characterized by higher levels of bridging social capital (third and fourth columns in the bottom part of Table 5). Importantly, as discussed in footnote 7, the measure of bridging social capital does not include religious groups, in line with the evidence provided by Deller et al. (2018). The positive impact of social capital is confirmed when considering non-profit organizations as a proxy for civic engagement (last two columns in the bottom part of Table 5). Additionally, in this case, areas with a greater density of non-profit organizations report a stronger relationship between crowdfunding and the number of employment-generating establishments.

Overall, the second hypothesis concerning the role of social capital as strengthening the effect of crowdfunding is confirmed. There are two exceptions, however. First, tight local connections seem to reduce the impact of crowdfunding. Assuming that clustering is driven by homophily (Chetty et al., 2022; McPherson et al., 2001), this result can be explained by tight networks among similar individuals preventing entrepreneurs from using crowdfunding or pursuing new ventures if it is perceived as being against local norms (de Vaan et al., 2019; Deller et al., 2018). Second, using better time-aligned data on social capital indicates possible downsides of social capital. The general measure of bridging and bonding social capital does not support the second hypothesis. A comparison of this measure with the measure of bridging social capital (without religious organizations) suggests, in line with the arguments of Deller et al. (2018), that (some) religious traditions may actually hinder entrepreneurial dynamics at the local level. Despite these two exceptions, the findings are well aligned with the literature and confirm how networks characterized by openness and more bridging nature facilitate innovative and entrepreneurial dynamics as opposed to strong network ties among similar individuals (Chetty et al., 2022; Cortinovis et al., 2017; Putnam, 2001).

### Robustness checks

I perform different checks to test the robustness of my findings. First, given the complexity of empirically identifying rural areas (Cattivelli, 2024; Ratcliffe et al., 2016), a first check (first two columns of Table 6) consists of assessing the validity of my findings to changes in the definition of rural areas. To this end, I build a new sample that considers rural counties with the characteristics suggested by Isserman (2005)^9^. As this classification does not include any measure of remoteness – recently emerged in the literature as an important aspect of rurality (Cattivelli, 2024; Cuéllar-Fernández et al., 2024; Kärnä & Stephan, 2022) – I further filter out counties that are less than 50 miles from an urban core. Using this alternative definition of rural areas increases the number of counties in the samples (836), mostly because of the higher threshold in terms of population density compared with the baseline definition. The results are overall in line with those discussed previously, with *CF (dummy)* being significant at the 10% level and *Large CF (dummy)* at the 5% level, even though the estimated coefficients are slightly smaller.^10^

**Table 6:** Robustness checks

	Alt. Rural	Alt. Rural	Remote	Remote	Highly Remote	Highly Remote	Most Remote	Most Remote
CF (dummy)	0.011 +		0.057*		0.003		0.019	
	(0.006)		(0.023)		(0.014)		(0.015)	
Large CF (dummy)		0.016*		0.088 +		0.014		0.063***
		(0.007)		(0.050)		(0.017)		(0.018)
Relatedness density	0.001*	0.001*	−0.000	−0.000	0.001 +	0.001 +	0.000	0.000
	(0.000)	(0.000)	(0.001)	(0.001)	(0.001)	(0.001)	(0.000)	(0.000)
Tot. employment (log)	0.164***	0.164***	0.104***	0.104***	0.144**	0.144**	0.194***	0.193***
	(0.026)	(0.026)	(0.028)	(0.028)	(0.053)	(0.053)	(0.048)	(0.048)
Tot. patent count	0.000	0.000	0.000**	0.000**	−0.000	−0.000	−0.000	−0.000
	(0.000)	(0.000)	(0.000)	(0.000)	(0.000)	(0.000)	(0.000)	(0.000)
GDP (log)	0.058***	0.058***	0.030*	0.030*	0.037**	0.037**	0.099***	0.099***
	(0.010)	(0.010)	(0.013)	(0.013)	(0.013)	(0.013)	(0.026)	(0.027)
Finance (log + 1)	−0.003	−0.003	−0.000	−0.000	−0.003	−0.003	−0.003	−0.003
	(0.002)	(0.002)	(0.005)	(0.005)	(0.003)	(0.003)	(0.002)	(0.002)
Bank deposits (log + 1)	0.001	0.001	0.005 +	0.005 +	−0.003	−0.003	0.003***	0.003***
	(0.001)	(0.001)	(0.003)	(0.003)	(0.003)	(0.003)	(0.001)	(0.001)
Num.Obs	246,572	246,572	67,652	67,652	55,876	55,876	27,729	27,729
Pseudo R2	0.694	0.694	0.663	0.663	0.635	0.635	0.595	0.595
NAICS3d-County FE	X	X	X	X	X	X	X	X
Year FE	X	X	X	X	X	X	X	X
Clustered SE	County	County	County	County	County	County	County	County

+ *p* < 0.1, * *p* < 0.05, ** *p* < 0.01, *** *p* < 0.001

Second, and related to the definition of my sample, I explore whether the results change with respect to the remoteness of the county. To this end, I divide the original samples into three groups: remote (between 50 and 75 miles from an urban core, columns 3 and 4 in Table 6), highly remote (between 75 and 100 miles, columns 5 and 6 in Table 6) and most remote (more than 100 miles away, columns 7 and 8 in Table 6). The impact of crowdfunding appears to be strongest for counties that are either remote or most remote, whereas it is not statistically significant for those between 75 and 100 miles. Comparing the size of the coefficients, the estimated coefficients in Table 6 appear larger than those in my baseline, possibly suggesting strongly localized effects. Interestingly, the coefficient for *CF (dummy)* is statistically significant at the 5% level, whereas the coefficient for *Large CF (dummy)* is 10% in the case of remote counties, whereas only *Large CF (dummy)* is strongly positive and significant (at the 0.1% level) in the most remote counties. This may suggest that counties most far away from urban areas rely more intensively on alternative sources of funding, as suggested by the literature on the geography of crowdfunding and entrepreneurial finance (Bernardino et al., 2016; Cumming et al., 2021; Lee & Brown, 2017; Ughetto et al., 2019).

Third, while all the regressors in the baseline model are lagged in time, it is relevant to rule out possible reverse causality issues. To this end, I add lead and lag values for my main variable of interest, *Large CF (dummy)*. As the first two columns of Table 7 show, only the one-year lagged variable produces a positive and significant coefficient, suggesting that pretrends or simultaneity do not drive my results.

**Table 7:** Robustness checks with lead-lag, placebo test and matched samples

	Lead-Lag	Lead-Lag	Fake CF	Matched PSM	Matched CEM
Large CF t + 2 (dummy)		0.007			
		(0.016)			
Large CF t + 1 (dummy)	0.032	0.045			
	(0.020)	(0.028)			
Large CF t + 0 (dummy)	0.018	0.013			
	(0.015)	(0.022)			
Large CF (dummy)	0.063***	0.062***			
	(0.011)	(0.018)			
Large CF t-2 (dummy)		0.020			
		(0.023)			
Large CF Fake (dummy)			0.008		
			(0.022)		
Large CF (dummy)				0.035*	0.032*
				(0.016)	(0.015)
Relatedness density	0.001	0.000	0.000	−0.000	−0.001
	(0.000)	(0.000)	(0.000)	(0.001)	(0.001)
Tot. employment (log)	0.149***	0.106**	0.147***	0.131 +	0.073 +
	(0.040)	(0.037)	(0.033)	(0.068)	(0.044)
Tot. patent count	−0.000	0.000	0.000	0.000	0.000
	(0.000)	(0.000)	(0.000)	(0.000)	(0.000)
GDP (log)	0.065***	0.059***	0.055***	0.005	0.045 +
	(0.017)	(0.014)	(0.014)	(0.036)	(0.026)
Finance (log + 1)	−0.001	−0.003	−0.001	−0.008*	−0.009**
	(0.003)	(0.003)	(0.003)	(0.004)	(0.003)
Bank deposits (log + 1)	0.002	0.041*	0.003**	0.002	0.002
	(0.001)	(0.019)	(0.001)	(0.005)	(0.004)
Num.Obs	126392	80642	151257	4608	14081
Pseudo R2	0.641	0.627	0.645	0.797	0.773
NAICS3d-County FE	X	X	X	X	X
Year FE	X	X	X	X	X
Clustered SE	County	County	County	County	County

The fourth and fifth robustness checks focus instead on reducing the concerns for endogeneity in the findings. In the context of a rather spatially concentrated phenomenon such as crowdfunding, it is important to check whether pre-existing heterogeneity may explain the use and impact of crowdfunding platforms. To this end, I first perform a placebo test randomly re-allocating *Large CF (dummy)* to other observations. The third column of Table 7 shows that the *Large CF Fake (dummy)* does not report a significant coefficient, as expected. This suggests that the impact on the number of establishments applies only when considering truly treated cases, and it is not driven by time trends or other types of noise in the data.

In addition, following Cuéllar-Fernández et al. (2024), my fifth set of robustness checks uses two different matching algorithms to create a balanced subsample of “treated” and “control” units. Specifically, the matching strategies I use are as follows: 1) a mix of propensity score and exact matching and 2) coarsened exact matching (CEM) (Ho et al., 2007, 2011). The matching exercises are based on different variables: relatedness density, the level of GDP of the county, the GDP contribution of the financial sector, the amount of deposits held in local banks and the number of employees in the county. I also require treated and control observations to be in the same state and year groups. Notably, for the matching exercise, I use information from the years between 2005 and 2008, before Kickstarter (and crowdfunding in general) became popular. This helps further ensure exogeneity. The choice of these two matching methods allows different samples to be built to assess both internal and external validity. As seen in Appendix 3, where Table 9 with the pre- and post-matching descriptives is reported, the first strategy leads to the best balancing, with effectively no significant difference between the means of the treated and control groups. However, as shown by the number of observations in the fourth column of Table 7, the subsample created is relatively small in size. Using CEM leads to the definition of a larger and overall balanced sample, which helps reduce concerns about limited external validity. However, in the CEM sample, the balance is not perfect,^11^ and the results based on these samples should be interpreted keeping this limitation in mind.

I use these samples to re-estimate the model with *Large CF (dummy)*, which is similar to a two-way fixed effect difference-in-differences method. However, the fact that I need to rely on nonlinear (Poisson) estimators for my dependent variable and that receiving crowdfunding is a relatively rare event poses technical issues in convincingly using difference-in-differences techniques. For this reason, robustness checks, even those pertaining to the matched samples, cannot be taken as establishing causal relationships. Nonetheless, these checks largely confirm the results of the baseline regressions and indicate that receiving crowdfunding is associated with an increase in the number of employment-generating establishments in the same industry‒county group the following year. The magnitude of the estimated effects are also in line with those of my baseline model, suggesting an increase of approximately 3% in the number of establishments in the county‒industry group.

## Conclusion

Entrepreneurship has been seen by policymakers and scholars alike as a possible means to stimulate innovation and economic development in rural areas, often characterized by structural weaknesses (Aguilar, 2021). While creating and sustaining a business in rural regions poses significant and well-known challenges (Huiban, 2011; Korsgaard et al., 2015), recent literature highlights that rural areas exploit specific resources and capabilities that stimulate local innovative and entrepreneurial dynamics (Castaldi et al., 2025). In this sense, rural entrepreneurial ecosystems rely less on size, infrastructure and technology and instead leverage alternative configurations of factors, especially those related to endogenous characteristics and external linkages (Mayer & Motoyama, 2020; Mayer et al., 2016; Meili & Shearmur, 2019). Nevertheless, one of the main challenges for rural entrepreneurs is obtaining access to capital to sustain their ventures (Cowling et al., 2021; Frimanslund, 2022; Lee & Brown, 2017). In this respect, crowdfunding platforms were heralded as a possible new way for rural entrepreneurs to gather funds and stimulate entrepreneurship (Schwartz, 2012). As discussed in the theoretical framework, crowdfunding may be especially important for rural entrepreneurs, as it contributes to addressing some of the weaknesses of rural EEs in terms of providing capital (Bernardino et al., 2016; Cumming et al., 2021; Muñoz & Kimmitt, 2019b) but also offers crucial insights and relevant knowledge (Agrawal et al., 2014; Elia et al., 2020; Hervé & Schwienbacher, 2018; Martínez-Climent et al., 2021). In addition, as personal social networks are crucial in crowdfunding (Butticè et al., 2017; Cai et al., 2021; Eiteneyer et al., 2019), high levels of social capital in rural areas may complement rural entrepreneurs’ ability to leverage opportunities on crowdfunding platforms (Giudici et al., 2018). However, the empirical evidence on crowdfunding and rural entrepreneurship has been rather limited (Bernardino et al., 2016; Cumming et al., 2021; Mollick & Kuppuswamy, 2016). This paper aims to provide a theoretical discussion and some evidence as to whether crowdfunding fosters entrepreneurial activity in rural areas.

After reviewing the literature and framing my work using entrepreneurial ecosystems as the main theoretical construct, I discuss possible mechanisms linking crowdfunding, social capital and entrepreneurial dynamics. Using fine-grained data on 3-digit NAICS industries across U.S. counties over the period 2009–2016, I find that the industry-location groups characterized by successful crowdfunding campaigns host a greater number of ventures after receiving funds. My main conclusions suggest that the successful use of crowdfunding is consistently positively associated with a greater number of ventures in the industry-location. Interestingly, local social capital conditions play an important role in the relationship between crowdfunding and the number of ventures. Specifically, when the sample is split, the impact of crowdfunding is strongest for areas characterized by high (bridging) social capital. This finding suggests that some typically rural characteristics (e.g., high social capital) may be important for fully exploiting and making use of new opportunities such as crowdfunding (Giudici et al., 2018). In terms of policy, these results are important for highlighting potential critical factors for successfully fostering entrepreneurial activities in rural areas.

I test the robustness of my results against a different possible definition of rural counties, and I perform a balancing exercise using propensity score and exact matching. Overall, my findings are confirmed. Moreover, some limitations remain, and future research should attempt to address these shortcomings more convincingly. First, the mechanisms highlighted in the literature and put forward by this paper, namely, the provision of capital resources and knowledge from the crowd, could not be explicitly tested in the settings of this paper. This is an important limitation, as this contribution does not pinpoint the exact link between crowdfunding and greater number of ventures. Second, while using within industry-location group variation to identify the impact of crowdfunding and the matching exercise reduce the concern for endogeneity, the causal relationship between crowdfunding and entrepreneurial dynamics could not be fully established. This represents an important limitation, as causal evidence would provide a more solid basis for designing suitable policy instruments. Third, with respect to the data used, this paper leverages information on a specific reward-based crowdfunding platform, Kickstarter. The focus on only one crowdfunding channel represents a limitation of the paper, since an entrepreneur may rely on additional or alternative sources. Relatedly, while the KIUS database is extensive and provides rather complete coverage (approximately 68% using global data, with the coverage being better for large campaigns and slightly worse for smaller ones; see the appendix for more information), some successful campaigns are likely to be missing from the data. While this is probably a relatively small share, incomplete coverage may bias the results, especially if the sample I have used includes more successful projects.

These limitations clearly point to possible research avenues for the future. In this respect, further research is needed to explore the possible mechanisms linking crowdfunding and the entry of new establishments better. From an empirical quantitative perspective, this requires leveraging more detailed data as well as developing methods (e.g., DiD) suitable or directly applicable to non-linear models. For example, while this analysis focuses on the short-term impact of crowdfunding, a longer time dimension would allow to capture longer-term effects and dynamics. These issues are particularly relevant since the full effect of crowdfunding may take longer than one year to materialize and may even change over time (e.g., if the campaign leads to the emergence of a local ecosystem or simply fades away). In addition, using comments from backers more systematically or building a more general database with information from multiple platforms may represent important steps forward in better understanding the relationship between crowdfunding and rural entrepreneurship. Moreover, taking a more qualitative approach is particularly important in future research. In-depth interviews and content analysis of crowdfunding platform interactions would help not only to complement the quantitative findings but also to provide richer insights into entrepreneurial processes and shed light on specific mechanisms, preferences, challenges and bottlenecks shaping the use and impact of crowdfunding in rural areas.

In addition, the role of social capital, which has been widely investigated in the crowdfunding literature, deserves more attention. As reported in this paper, and in line with other contributions (Deller et al., 2018), the mediating role of social capital is not unambiguously positive (a high level of clustering has a negative impact), and it also appears to be contingent on specific types of groups (bridging-type organizations but not religious groups). Additionally, in this respect, combining insights from both quantitative and qualitative research will be important to better grasp how local social structures affect entrepreneurial dynamics and what role these structures play in rural entrepreneurial ecosystems. Relatedly, an issue deserving further attention is the generalizability of our findings on the impacts of crowdfunding. While rural entrepreneurs across the globe often face similar challenges in accessing finance, rural areas also differ in institutional settings, development of financial markets and social capital, potentially making the conclusions of our analysis less applicable to other geographical contexts. From this perspective, future research could extend this analysis to non-U.S. contexts to examine how variations in institutional and financial environments shape the effects of crowdfunding on new venture emergence in rural areas.

## Figures and Tables

**Fig. 2:**
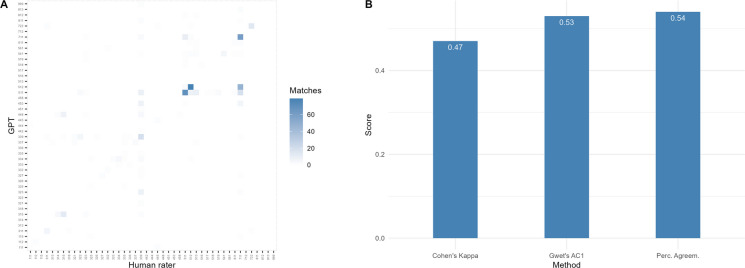
Data validation comparing human and machine

**Fig. 3:**
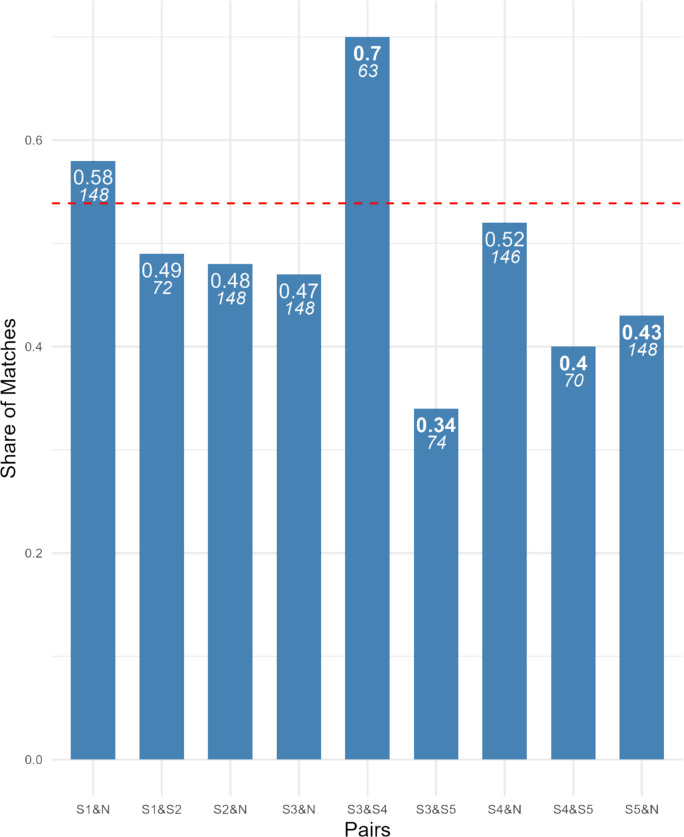
Data validation comparing the human rater (N) and students

**Fig. 4:**
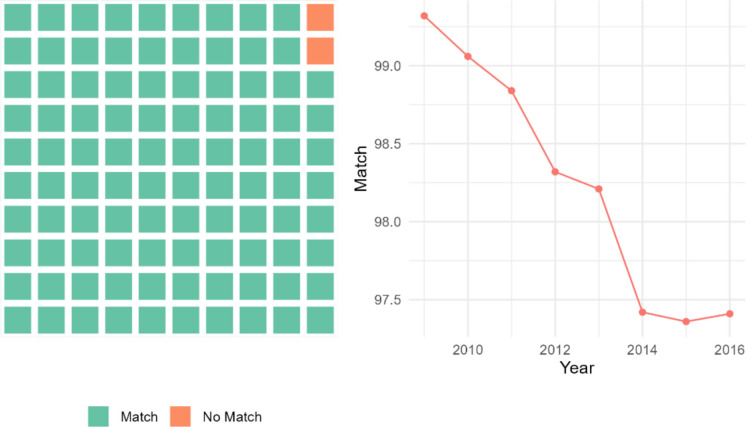
Accuracy of the generated data

## Data Availability

The KIUS dataset analysed during the current study are available in the Yoda repository, https://public.yoda.uu.nl/geo/UU01/14B0U6.html

## Appendix 1: The KIUS database

For this project, I used AI (chatGPT 3.5 Turbo) to classify approximately 300,000 Kickstarter projects into different industry codes. Before the data are used, it is crucial to discuss the data development procedure and data validation performed. In terms of data development, the chatGPT API was provided with the title, blurb (short introductory description of the project under the project's title) and the relevant category and subcategory in which the project is listed. The algorithm was then asked, on the basis of this information, to classify the project into one of the 311 4-digit codes of the 2017 NAICS classification. The process used to develop the data is more thoroughly discussed by Mitra et al. (2024), and the python script used to develop the data is available in this GitHub repository: https://github.com/UtrechtUniversity/generative-ai. Using the information developed with the help of AI, the information at the KS project level has been aggregated by county, year and 3-digit NAICS codes. Data validation was also performed on the basis of 3-digit data.

Fig. 2.

With respect to data validation, we perform four main tests. First, we check the reliability of the prediction made by ChatGPT (version 3.5, produced in September 2023) and approach the issue as an interrater reliability question (Gwet, 2014; Klein, 2018). Essentially, we consider ChatGPT as a possible rater facing a classification problem and compare its performance to that of other human rater. To this end, given the impossibility of rating nearly 300,000 projects, I created a sample of 500 projects, stratified according to the distribution of Kickstarter categories to maximize the representativity of the sample. I then proceed and classify each of the projects into the best-fitting 3-digit code out of the 86 possible codes of the NAICS 2017 classification. Importantly, as a human rater, I manually selected the NAICS code independently from ChatGPT (i.e., without knowing what code the algorithm selected) and used the same information available to ChatGPT (title, blurb, category and subcategory). I then calculate some basic descriptive statistics (Fig. 2), in particular a confusion matrix (Panel A). I also computed two standard measures of reliability across raters: Cohen’s Kappa and Gwet’s AC1 and percentage agreement (Panel B). With respect to the confusion matrix, it is interesting to note that the machine and I mostly agree, with the main exception being for cases in which ChatGPT provided the code 511 and the human rater code 711. A data inspection indicates that these projects are creative projects—in particular music album production—which are inherently difficult to classify, as they both imply some music performance (NAICS code 711) as well as the publication of the album (NAICS code 511). Concerning the graph in Panel B, in 54% of the cases, the human and ChatGPT perfectly agreed on the same code (the diagonal in the confusion matrix), with both Cohen’ Kappa and Gwet’s AC1 suggesting a moderate level of agreement. While the moderate agreement may suggest that we can only partially trust the data developed, it is important to stress the complexity of the task, given the number of classes among which to choose (86 at the 3-digit level).

To obtain a more credible benchmark against which to evaluate the score, I performed a second test to determine whether different humans would agree more or less than the human and chatGPT would agree. To this end, 5 third-year economics bachelor’s degree students were asked to match a list of projects to the relevant 2017 NAICS codes. Each student had a specific set of approximately 140 projects, which were randomly drawn from the same sample of hand-coded projects. The sample each student had been built in such a way to have at least some overlap with the sample of someone else. Figure 2. A above reports the share of overlap between different pairs of students and between students and human rater (N), including both the share of agreement (number on the top, in bold if statistically different from 0.54, the share of agreement between the human rater and the ChatGPT) and the amount of total sample size (i.e., the number of projects present in both samples, in italics; for instance, the S1 and S2 samples had 72 commons projects, and the two students agreed in 49% of the cases). The red dashed line represents the overlap between the human rater and ChatGPT (0.54). The highest level of overlap reached is between students S3 and S4 (70% of the cases of 63 projects evaluated), and the lowest is between students S3 and S5 (34% of the cases out of 74 projects). In both cases, together with S4 and S5 and S5 and N, the share of matches recorded is significantly different (p value < 0.05) from that between the human rater (N) and the GPT. This comparison shows that in the classification task, chat GPT, while not the top performer, did not perform better than humans did. In only one case and the one with the fewest projects, humans performed better in a statistically significant way. Overall, this provides more confidence in the quality of the developed database.

Fig. 3.

While the previous two validation tests (human rater vs. machine, comparison between human vs. machine and human vs. human) aimed at checking how accurate chatGPT was, the last two tests aimed at checking whether the data developed provided a plausible picture. In other words, I want to check whether the industry-location-year groups identified by ChatGPT as receiving crowdfunding resources effectively exist in the official data. For this reason, rather than focusing on all 300,000 projects classified (which cover the period up to 2022), these checks focus on the period 2009–2016, which pertain to my analysis.

Fig. 4.

To this end, I compare the data I developed with the County Business Patterns database (Eckert et al., 2020). Figure 4 above reports my findings. Overall, in approximately 98% of the cases, the industry-county-year group to which a project was allocated existed in the official data, as shown by the waffle graph in Panel A. Checking the accuracy of the data over the years of my sample, we see in Panel B a slight decline over time, with the overall consistency never falling below 97%.

The second and last check on the accuracy of the data focuses on the coverage in terms of Kickstarter. In particular, the objective of this check is to assess how many of the projects officially reported on Kickstarter are effectively in the database. Unfortunately, Kickstarter did not provide (not even when asked) more detailed information in terms of the time and geographical breakdown of projects. However, the general “Stats” page on Kickstarter provides information in terms of the number of projects funded, the amount raised and a breakdown by size of the project on a global scale. Using waybackmachine, I checked the coverage of my sample and focused on the US in the period 2009–2016 against the available information. Compared with the global data available on January 1 st, 2017, the database covers 68% of the 117,813 projects funded and 62% of the total funding back then (2,806 million USD). Table 8 below compares the databases with the available breakdown in terms of the size of the projects, confirming good coverage. While a slightly greater proportion of small projects are missed (less than 1,000 USD), at least 70% of the projects in the larger categories are included in the KIUS database.

**Table 8:** Comparing data used with official global Kickstarter statistics

Size group (in USD)	Succ. proj. on KS stats	Succ. proj. in KIUS	Coverage
< 1 k	14,101	8948	0.63
1 k < = x < 10 k	67,155	46,996	0.7
10 k < = x < 20 k	16,889	12,233	0.72
20 k < = x < 100 k	16,008	11,563	0.72
100 k < = x < 1 M	3450	2418	0.7
x < = 1 M	210	167	0.8

## Appendix 2: Relatedness and relatedness density

The economic geography literature has since long recognized the importance of cognitive proximity and knowledge recombination in shaping the composition of local economies (Boschma, 2005). A method that has become popular in this literature and that allows to estimate how similar two industries are is the relatedness framework developed by Hidalgo et al. (2007). Specifically, by calculating in which activities (industries, products, etc.) an area has a comparative advantage and studying how frequently two specializations occur simultaneously, it is possible to estimate how related two industries or products are (Hidalgo et al., 2007). While empirically driven, the method theoretically considers that the capabilities required to operate in an industry (e.g., shoe manufacturing) may be similar (related) to those also required by other industries (e.g., leather bag manufacturing) and rather different from others (e.g., aerospace). From this perspective, a firm starting to produce leather bags in a region already specialized in shoe manufacturing can take advantage of the local availability of relevant capabilities (for a more general introduction, please see Hidalgo et al., 2018). Effectively, this method can therefore be used to capture how related a certain industry is to industries already present locally, which is something that the literature on EE has recently highlighted as potentially important to consider (Audretsch & Belitski, 2021).

To estimate relatedness and capture the proximity of each industry to the local specialization, I follow what has become a rather standard method in economic geography (Balland, 2017; Cortinovis et al., 2017; Hidalgo et al., 2018). First, I compute the location quotient for each county and industry in every year:

$${LQ}_{i,c,t}=\frac{{E}_{i,c,t}/\sum_{i}{E}_{i,c,t}}{\sum_{c}{E}_{i,c,t}/\sum_{i,c}{E}_{i,c,t}}$$

where the numerator represents the share of employment ${E}_{i,c,t}$ in industry *i*, county *c*, year *t* over total employment in county *c*, in year *t*, whereas the denominator is the share of employment in industry *i* in year *t* in the whole US (summation is over all the counties) over total employment in the US (summation is over all counties and industries). Once the location quotient is computed, I apply a bootstrapping method to compute the industry-specific threshold to define a binary variable ${spec}_{i,c,t}$ indicating whether a specific county specializes in a certain industry (Cortinovis et al., 2017, 2020; Pominova et al., 2022; Qiao et al., 2024; Tian, 2013).

The yearly vectors of location-industry specializations are then used to compute relatedness. The measure of relatedness is calculated as the ratio between the observed cooccurrences in specializations between industry *i* and industry* j* and a random benchmark. In formal terms:

$${\varphi }_{i,j,t}=\frac{{c}_{i,j,t}}{\left[\left(\frac{{S}_{i,t}}{{T}_{t}}\right)*\left(\frac{{S}_{j,t}}{{T}_{t}-{S}_{i,t}}\right)+\left(\frac{{S}_{j,t}}{{T}_{t}}\right)*\left(\frac{{S}_{i,t}}{{T}_{t}-{S}_{j,t}}\right)\right]*\left(\frac{{T}_{t}}{2}\right)}$$

In the equation above, ${c}_{i,j,t}$ represents the co-occurrence count of specializations in sectors *i* and *j* in year *t*, ${S}_{i,t}$ and ${S}_{j,t}$ are the total number of occurrences of each of the two industries, respectively, and ${T}_{t}$ is the total number of occurrences of any sector. Essentially, ${\varphi }_{i,j,t}$ is the ratio between the count number of cases (counties) in which the specializations in i and j simultaneously occur (nominator) and the number of counties in which we would expect the specializations to cooccur in random settings (denominator). In other words, ${\varphi }_{i,j}$ takes a value of 1 when the co-occurrence found in the data is as frequent as at random, whereas it carries a higher (lower) weight when co-occurrence occurs more (less) frequently. We further modify the matrix $\Phi$ such that 0 is the value of ${\varphi }_{i,j,t}$, which is equal to or less than 1, since their relatedness is coincidental. The matrix is then used to construct a density indicator as in Hidalgo et al. (2007). To do that, I computed the sum for specializations in industry *j* related to industry *i* in the same county *c* at time *t*, weighted by the pairwise relatedness score (${\varphi }_{i,j,t}$). I then divide this score by the theoretically possible industries related to *i*, effectively obtaining a ratio between the observed related specializations present in county *c* at time *t* and the maximum possible level (i.e., if all the related specializations were present locally).

$${density}_{i,c,t}=\frac{\sum_{k\ne i}{\varphi }_{i,j,t}*{spec}_{j,c,t}}{\sum_{k\ne i}{\varphi }_{i,j,t}}$$

Intuitively, the measure of relatedness density captures how close in terms of capabilities the focal industry (*i*) is to all the related industries (*j*) in which the county *c* is already specialized at time *t*. The R package EconGeo (Balland, 2017) provides a good introduction to the method, which has been widely used in the literature (see Hidalgo et al., 2018).

## Appendix 3: Results of the matching exercises

Table 9.

**Table 9:** Results of the matching exercises

Variable	Mean Treated	Mean Controls	T Statistic	p-value	Sample
# Establishments	12.97	5.23	4.05	0	Full sample
Density	5.9	8.64	-6.78	0	Full sample
Tot. employment (log)	8.48	7.31	18.87	0	Full sample
Tot. patent count	16.17	2.04	5.92	0	Full sample
GDP (log)	13.2	12.21	18	0	Full sample
Finance (log1p)	11.58	10.41	13.48	0	Full sample
Bank deposits (log1p)	12.29	11.5	7.06	0	Full sample
# Establishments	12.97	10.52	1.22	0.22	Matched sample (PSM)
Density	5.9	5.76	0.3	0.77	Matched sample (PSM)
Tot. employment (log)	8.48	8.46	0.26	0.79	Matched sample (PSM)
Tot. patent count	16.17	15.77	0.15	0.88	Matched sample (PSM)
GDP (log)	13.2	13.22	-0.4	0.69	Matched sample (PSM)
Finance (log1p)	11.58	11.56	0.21	0.83	Matched sample (PSM)
Bank deposits (log1p)	12.29	12.25	0.34	0.74	Matched sample (PSM)
# Establishments	12.56	10.15	1.25	0.21	Matched sample (CEM)
Density	5.58	3.15	6.31	0	Matched sample (CEM)
Tot. employment (log)	8.49	8.44	0.79	0.43	Matched sample (CEM)
Tot. patent count	16.26	12.9	1.36	0.18	Matched sample (CEM)
GDP (log)	13.21	13.1	2.01	0.05	Matched sample (CEM)
Finance (log1p)	11.58	11.5	0.93	0.35	Matched sample (CEM)
Bank deposits (log1p)	12.3	12.4	-0.86	0.39	Matched sample (CEM)

## Footnotes

1 In the text, I refer to ventures and establishments interchangeably, with the empirical analysis focusing on the number of employment-generating establishments reported in the County Business Patterns database.

2 It is important to notice that missing values do not affect the dependent variable, as the number of employment-generating establishments is consistently reported in the period of analysis. The use of the data developed by Eckert et al. (2020) is nonetheless important for obtaining better coverage in control variables and using data with harmonized NAICS codes. As explained by the developers of the data, from 2017 onward the Census Bureau has changed its suppression protocol and started omitting cells with fewer than 3 establishments.

3 To compute the total deposit by county, I consider only physical banks (e.g. no cyber offices) with either full banking service or limited services focused on loans. Specifically, I include establishments coded in the FDIC data as: Full Service, brick and mortar office; Full Service, retail office; Limited Service, loan production office; Limited Service, consumer credit office.

4 The choice of not including demographic aspects in the classification of this paper was deliberate, as it would introduce an important risk of selection bias (areas with higher average age would be less entrepreneurial by definition).

5 It should be noticed that the CBP focuses on employment-generating businesses, effectively excluding self-employed entrepreneurs. The use of a measure capturing more established business activities represents a limitation of this study.

6 While the paper was originally published in 2006, the authors have updated their data up to 2009. The data is available here: https://nercrd.psu.edu/data-resources/county-level-measure-of-social-capital/social-capital-variables-for-1997-2005-and-2009/.

7 Importantly, I have excluded participation in religious organizations when computing the measure of bridging social capital. This choice is motivated by the fact that, in the specific context of entrepreneurship, recent evidence suggests that religious organizations hamper entrepreneurial activities (Avnimelech & Zelekha, 2023; Deller et al., 2018). Including religious organizations in bridging social capital leads to similar results as those for the general social capital variable and are available upon request. The other variables are used as provided by the authors (Rupasingha et al., 2006).

8 The data reported is official data from the Census Bureau, freely available through the “usdata” R package (Çetinkaya-Rundel et al., 2020/2024).

9 Accordingly, to be considered rural a county must have a population density below 500 people per square mile, and either 90% or more of its residents must live in rural areas or less than 10,000 living in urban areas.

10 The smaller coefficients do not affect the significant of the results, except for *Amount raises (000 s)*, which is no longer statistically significant (result available upon request).

11 As shown in Table 8 in the Appendix, using CEM improves the balance compared to the full sample, even though the difference in means between treated and control groups are significant for two variables, *Relatedness density* and *GDP (log)*.

## References

[CR1] Acs, Z. J., & Kallas, K. (2008). State of literature on small- to medium-sized enterprises and entrepreneurship in low-income communities. In G. Yago, J. R. Barth, & B. Zeidman (Eds.), *Entrepreneurship in Emerging Domestic Markets* (pp. 21–45). Springer. 10.1007/978-0-387-72857-5_3

[CR2] Acs, Z. J., Audretsch, D. B., & Lehmann, E. E. (2013). The knowledge spillover theory of entrepreneurship. *Small Business Economics,**41*(4), 757–774. 10.1007/s11187-013-9505-9

[CR3] Agrawal, A., Catalini, C., & Goldfarb, A. (2014). Some simple economics of crowdfunding. *Innovation Policy and the Economy,**14*, 63–97. 10.1086/674021

[CR4] Agrawal, A., Catalini, C., & Goldfarb, A. (2015). Crowdfunding: Geography, social networks, and the timing of investment decisions. *Journal of Economics & Management Strategy,**24*(2), 253–274. 10.1111/jems.12093

[CR5] Aguilar, E. C. (2021). Rural entrepreneurial ecosystems: A systematic literature review for advancing conceptualisation. *Entrepreneurial Business and Economics Review,**9*(4), Article 4. 10.15678/EBER.2021.090407

[CR6] Almeida, J., & Daniel, A. D. (2025). Addressing the distinctive features of entrepreneurial ecosystems in low-density territories. *Journal of Entrepreneurship and Public Policy*. 10.1108/JEPP-07-2023-0069

[CR7] Alsos, G. A., Carter, S., & Ljunggren, E. (2014). Kinship and business: How entrepreneurial households facilitate business growth. *Entrepreneurship & Regional Development,**26*(1–2), 97–122. 10.1080/08985626.2013.870235

[CR8] Anderson, A. R. (2000). Paradox in the periphery: An entrepreneurial reconstruction? *Entrepreneurship & Regional Development,**12*(2), 91–109. 10.1080/089856200283027

[CR9] Audretsch, D. B., & Belitski, M. (2017). Entrepreneurial ecosystems in cities: Establishing the framework conditions. *The Journal of Technology Transfer,**42*(5), 1030–1051. 10.1007/s10961-016-9473-8

[CR10] Audretsch, D. B., & Belitski, M. (2021). Towards an entrepreneurial ecosystem typology for regional economic development: The role of creative class and entrepreneurship. *Regional Studies,**55*(4), 735–756. 10.1080/00343404.2020.1854711

[CR11] Avnimelech, G., & Zelekha, Y. (2023). Religion and the gender gap in entrepreneurship. *International Entrepreneurship and Management Journal,**19*(2), 629–665. 10.1007/s11365-023-00855-4

[CR12] Balland, P. A. (2017). *Economic Geography in R: Introduction to the EconGeo Package* (SSRN Scholarly Paper 2962146). 10.2139/ssrn.2962146

[CR13] Banfield, E. C. (with Internet Archive). (1958). *The moral basis of a backward society*. Free Press. http://archive.org/details/moralbasisofback00banf

[CR14] Basu, E.-M., Lindstrand, A., & Fichtel, J. (2025). Pushing the boundaries of entrepreneurial ecosystems: Antecedents to international network activity of entrepreneurial firms. *Small Business Economics*. 10.1007/s11187-025-01023-4

[CR15] Baù, M., Chirico, F., Pittino, D., Backman, M., & Klaesson, J. (2019). Roots to grow: Family firms and local embeddedness in rural and urban contexts. *Entrepreneurship Theory and Practice,**43*(2), 360–385. 10.1177/1042258718796089

[CR16] Belleflamme, P., Lambert, T., & Schwienbacher, A. (2014). Crowdfunding: Tapping the right crowd. *Journal of Business Venturing,**29*(5), 585–609. 10.1016/j.jbusvent.2013.07.003

[CR17] Bergé, L. R. (2018). Efficient estimation of maximum likelihood models with multiple fixed-effects: The R package FENmlm. *CREA Discussion Paper*, *2018*(13).

[CR18] Bernardino, S., Freitas Santos, J., & Cadima Ribeiro, J. (2016). Social crowdfunding: A new model for regional development? *Journal of Urban and Regional Analysis*, *8*(2). 10.37043/JURA.2016.8.2.1

[CR19] Block, J. H., Colombo, M. G., Cumming, D. J., & Vismara, S. (2018). New players in entrepreneurial finance and why they are there. *Small Business Economics,**50*(2), 239–250. 10.1007/s11187-016-9826-6

[CR20] Boschma, R. (2005). Proximity and innovation: A critical assessment. *Regional Studies,**39*(1), 61–74. 10.1080/0034340052000320887

[CR21] Breznitz, S. M., & Noonan, D. S. (2020). Crowdfunding in a not-so-flat world. *Journal of Economic Geography,**20*(4), 1069–1092. 10.1093/jeg/lbaa008

[CR22] Butticè, V., Colombo, M. G., & Wright, M. (2017). Serial crowdfunding, social capital, and project success. *Entrepreneurship Theory and Practice,**41*(2), 183–207. 10.1111/etap.12271

[CR23] Cai, W., Polzin, F., & Stam, E. (2021). Crowdfunding and social capital: A systematic review using a dynamic perspective. *Technological Forecasting and Social Change,**162*, Article 120412. 10.1016/j.techfore.2020.120412

[CR24] Camilleri, M. A., & Bresciani, S. (2022). Crowdfunding small businesses and startups: A systematic review, an appraisal of theoretical insights and future research directions. *European Journal of Innovation Management,**27*(7), 2183–2209. 10.1108/EJIM-02-2022-0060

[CR25] Castaldi, C., Cortinovis, N., & Tessarin, M. S. (2025). Out of sight? Revealing creativity-led innovation in rural regions. *Papers in Evolutionary Economic Geography (PEEG)*.

[CR26] Cattivelli, V. (2024). Where is the city? Where is the countryside? Assessing the methods for the classification of urban, rural, and intermediate areas in Europe. *Journal of Rural Studies,**109*, Article 103288. 10.1016/j.jrurstud.2024.103288

[CR27] Çetinkaya-Rundel, M., Diez, D., & Dorazio, L. (2024). *usdata: Data on the States and Counties of the United States* [R]. OpenIntro. https://github.com/OpenIntroStat/usdata (Original work published 2020)

[CR28] Chetty, R., Jackson, M. O., Kuchler, T., Stroebel, J., Hendren, N., Fluegge, R. B., Gong, S., Gonzalez, F., Grondin, A., Jacob, M., Johnston, D., Koenen, M., Laguna-Muggenburg, E., Mudekereza, F., Rutter, T., Thor, N., Townsend, W., Zhang, R., Bailey, M., & Wernerfelt, N. (2022). Social capital I: Measurement and associations with economic mobility. *Nature,**608*(7921), 108–121. 10.1038/s41586-022-04996-4 35915342 10.1038/s41586-022-04996-4 PMC9352590

[CR29] Colombo, M. G., Franzoni, C., & Rossi–Lamastra, C. (2015). <article-title update="added">Internal social capital and the attraction of early contributions in crowdfunding. *Entrepreneurship Theory and Practice,**39*(1), 75–100. 10.1111/etap.12118

[CR30] Conroy, T., & Low, S. A. (2022). Entrepreneurship, broadband, and gender: Evidence from establishment births in rural America. *International Regional Science Review,**45*(1), 3–35. 10.1177/01600176211018749

[CR31] Cortinovis, N., Xiao, J., Boschma, R., & van Oort, F. G. (2017). Quality of government and social capital as drivers of regional diversification in Europe. *Journal of Economic Geography,**17*(6), 1179–1208. 10.1093/jeg/lbx001

[CR32] Cortinovis, N., Crescenzi, R., & van Oort, F. (2020). Multinational enterprises, industrial relatedness and employment in European regions. *Journal of Economic Geography,**20*(5), 1165–1205. 10.1093/jeg/lbaa010

[CR33] Cowling, M., Brown, R., & Lee, N. (2021). The geography of business angel investments in the UK: Does local bias (still) matter? *Environment and Planning a, Economy and Space,**53*(5), 1180–1200. 10.1177/0308518X20984484

[CR34] Cuéllar-Fernández, B., Fuertes-Callén, Y., & Serrano-Magdalena, A. (2024). Factors behind the resilience of rural startups. *Technological Forecasting and Social Change,**206*, Article 123521. 10.1016/j.techfore.2024.123521

[CR35] Cumming, D., Meoli, M., & Vismara, S. (2021). Does equity crowdfunding democratize entrepreneurial finance? *Small Business Economics,**56*(2), 533–552. 10.1007/s11187-019-00188-z

[CR36] de Vaan, M., Frenken, K., & Boschma, R. (2019). The downside of social capital in new industry creation. *Economic Geography,**95*(4), 315–340. 10.1080/00130095.2019.1586434

[CR37] del Olmo-García, F., Domínguez-Fabián, I., Crecente-Romero, F. J., & del Val-Núñez, M. T. (2023). Determinant factors for the development of rural entrepreneurship. *Technological Forecasting and Social Change,**191*, Article 122487. 10.1016/j.techfore.2023.122487

[CR38] Deller, S., Conroy, T., & Markeson, B. (2018). Social capital, religion and small business activity. *Journal of Economic Behavior & Organization,**155*, 365–381. 10.1016/j.jebo.2018.09.006

[CR39] Deller, S., Whitacre, B., & Conroy, T. (2022). Rural broadband speeds and business startup rates. *American Journal of Agricultural Economics,**104*(3), 999–1025. 10.1111/ajae.12259

[CR40] Dunkley, E. (2016, February 9). Crowdfunding rides to the rescue of many SMEs. *Financial Times*. https://www.ft.com/content/a1b69b96-475b-11e5-af2f-4d6e0e5eda22

[CR41] Duranton, G., & Puga, D. (2001). Nursery cities: Urban diversity, process innovation, and the life cycle of products. *American Economic Review,**91*(5), 1454–1477. 10.1257/aer.91.5.1454

[CR42] Eckert, F., Fort, T., Schott, P., & Yang, N. (2020). *Imputing Missing Values in the US Census Bureau’s County Business Patterns* (w26632; p. w26632). National Bureau of Economic Research. 10.3386/w26632

[CR43] Eiteneyer, N., Bendig, D., & Brettel, M. (2019). Social capital and the digital crowd: Involving backers to promote new product innovativeness. *Research Policy,**48*(8), Article 103744. 10.1016/j.respol.2019.01.017

[CR44] Eldridge, D., Nisar, T. M., & Torchia, M. (2021). What impact does equity crowdfunding have on SME innovation and growth? An empirical study. *Small Business Economics,**56*(1), 105–120. 10.1007/s11187-019-00210-4

[CR45] Elia, G., Margherita, A., & Passiante, G. (2020). Digital entrepreneurship ecosystem: How digital technologies and collective intelligence are reshaping the entrepreneurial process. *Technological Forecasting and Social Change,**150*, Article 119791. 10.1016/j.techfore.2019.119791

[CR46] Fanjul, A. P., Herrera, L., & Munoz-Doyague, M. F. (2023). Fostering rural entrepreneurship: An ex-post analysis for Spanish municipalities. *Technological Forecasting and Social Change,**197*, Article 122915. 10.1016/j.techfore.2023.122915

[CR47] Figueroa-Armijos, M., & Berns, J. P. (2022). Vulnerable populations and individual social responsibility in prosocial crowdfunding: Does the framing matter for female and rural entrepreneurs? *Journal of Business Ethics,**177*(2), 377–394. 10.1007/s10551-020-04712-0

[CR48] Figueroa-Armijos, M., & Johnson, T. G. (2013). Entrepreneurship in rural America across typologies, gender and motivation. *Journal of Developmental Entrepreneurship,**18*(02), Article 1350014. 10.1142/S1084946713500143

[CR49] Figueroa-Armijos, M., Dabson, B., & Johnson, T. G. (2012). Rural Entrepreneurship in a Time of Recession. *Entrepreneurship Research Journal*, *2*(1). 10.2202/2157-5665.1044

[CR50] Frimanslund, T. (2022). Financial entrepreneurial ecosystems: An analysis of urban and rural regions of Norway. *International Journal of Global Business and Competitiveness,**17*(1), 24–39. 10.1007/s42943-022-00050-2

[CR51] Frimanslund, T., Kwiatkowski, G., & Oklevik, O. (2023). The role of finance in the literature of entrepreneurial ecosystems. *European Planning Studies,**31*(2), 372–391. 10.1080/09654313.2022.2055962

[CR52] Garrod, B., Wornell, R., & Youell, R. (2006). Re-conceptualising rural resources as countryside capital: The case of rural tourism. *Journal of Rural Studies,**22*(1), 117–128. 10.1016/j.jrurstud.2005.08.001

[CR53] Giudici, G., Guerini, M., & Rossi-Lamastra, C. (2018). Reward-based crowdfunding of entrepreneurial projects: The effect of local altruism and localized social capital on proponents’ success. *Small Business Economics,**50*(2), 307–324. 10.1007/s11187-016-9830-x

[CR54] Glaeser, E. L., Kallal, H. D., Scheinkman, J. A., & Shleifer, A. (1992). Growth in cities. *Journal of Political Economy,**100*(6), 1126–1152. 10.1086/261856

[CR55] Grilli, L. (2019). There must be an angel? Local financial markets, business angels and the financing of innovative start-ups. *Regional Studies,**53*(5), 620–629. 10.1080/00343404.2018.1479524

[CR56] Gwet, K. L. (2014). *Handbook of inter-rater reliability: The definitive guide to measuring the extent of agreement among raters* (Fourth edition). Advances Analytics, LLC.

[CR57] Henderson, V., Kuncoro, A., & Turner, M. (1995). Industrial Development in Cities. *Journal of Political Economy,**103*(5), 1067–1090. 10.1086/262013

[CR58] Hervé, F., & Schwienbacher, A. (2018). Crowdfunding and Innovation. *Journal of Economic Surveys,**32*(5), 1514–1530. 10.1111/joes.12274

[CR59] Hidalgo, C. A., Klinger, B., Barabási, A.-L., & Hausmann, R. (2007). The product space conditions the development of nations. *Science,**317*(5837), 482–487. 10.1126/science.1144581 17656717 10.1126/science.1144581

[CR60] Hidalgo, C. A., Balland, P.-A., Boschma, R., Delgado, M., Feldman, M., Frenken, K., Glaeser, E., He, C., Kogler, D. F., Morrison, A., Neffke, F., Rigby, D., Stern, S., Zheng, S., & Zhu, S. (2018). The Principle of Relatedness. In A. J. Morales, C. Gershenson, D. Braha, A. A. Minai, & Y. Bar-Yam (Eds.), *Unifying Themes in Complex Systems IX* (pp. 451–457). Springer International Publishing. 10.1007/978-3-319-96661-8_46

[CR61] Ho, D., Imai, K., King, G., & Stuart, E. A. (2007). Matching as nonparametric preprocessing for reducing model dependence in parametric causal inference. *Political Analysis,**15*(3), 199–236. 10.1093/pan/mpl013

[CR62] Ho, D., Imai, K., King, G., & Stuart, E. A. (2011). MatchIt: Nonparametric preprocessing for parametric causal inference. *Journal of Statistical Software,**42*, 1–28. 10.18637/jss.v042.i08

[CR63] Huiban, J.-P. (2011). The spatial demography of new plants: Urban creation and rural survival. *Small Business Economics,**37*(1), 73–86. 10.1007/s11187-009-9228-0

[CR64] Isserman, A. M., Feser, E., & Warren, D. E. (2009). Why some rural places prosper and others do not. *International Regional Science Review,**32*(3), 300–342. 10.1177/0160017609336090

[CR65] Kärnä, A., & Stephan, A. (2022). Do firms in rural regions lack access to credit? Local variation in small business loans and firm growth. *Regional Studies,**56*(11), 1919–1933. 10.1080/00343404.2021.2016681

[CR66] Kim, K., & Viswanathan, S. (2019). The experts in the crowd: The role of experienced investors in a crowdfunding market. *MIS Quarterly,**43*(2), 347–372. 10.25300/MISQ/2019/13758

[CR67] Klein, D. (2018). Implementing a general framework for assessing interrater agreement in Stata. *The Stata Journal,**18*(4), 871–901. 10.1177/1536867X1801800408

[CR68] Knack, S., & Keefer, P. (1997). Does Social Capital Have an Economic Payoff? A cross-country investigation*. *The Quarterly Journal of Economics,**112*(4), 1251–1288. 10.1162/003355300555475

[CR69] Korsgaard, S., Ferguson, R., & Gaddefors, J. (2015). The best of both worlds: How rural entrepreneurs use placial embeddedness and strategic networks to create opportunities. *Entrepreneurship & Regional Development,**27*(9–10), 574–598. 10.1080/08985626.2015.1085100

[CR70] Kuppuswamy, V., & Bayus, B. L. (2015). *A Review of Crowdfunding Research and Findings*. Edward Elgar Publishing.

[CR71] Kuppuswamy, V., & Bayus, B. L. (2018). Crowdfunding creative ideas: The dynamics of project backers. In D. Cumming & L. Hornuf (Eds.), *The economics of crowdfunding: Startups, portals and investor behavior* (pp. 151–182). Springer International Publishing. 10.1007/978-3-319-66119-3_8

[CR72] Lang, R., & Fink, M. (2019). Rural social entrepreneurship: The role of social capital within and across institutional levels. *Journal of Rural Studies,**70*, 155–168. 10.1016/j.jrurstud.2018.03.012

[CR73] Lee, N., & Brown, R. (2017). Innovation, SMEs and the liability of distance: The demand and supply of bank funding in UK peripheral regions. *Journal of Economic Geography,**17*(1), 233–260. 10.1093/jeg/lbw011

[CR74] Martínez-Climent, C., Mastrangelo, L., & Ribeiro-Soriano, D. (2021). The knowledge spillover effect of crowdfunding. *Knowledge Management Research & Practice,**19*(1), 106–116. 10.1080/14778238.2020.1768168

[CR75] Mayer, H., & Motoyama, Y. (2020). Entrepreneurship in small and medium-sized towns. *Entrepreneurship & Regional Development,**32*(7–8), 467–472. 10.1080/08985626.2020.1798556

[CR76] Mayer, H., Habersetzer, A., & Meili, R. (2016). Rural–urban linkages and sustainable regional development: The role of entrepreneurs in linking peripheries and centers. *Sustainability,**8*(8), Article 8. 10.3390/su8080745

[CR77] McPherson, M., Smith-Lovin, L., & Cook, J. M. (2001). Birds of a feather: Homophily in Social Networks. *Annual Review of Sociology,**27*(1), 415–444. 10.1146/annurev.soc.27.1.415

[CR78] Meili, R., & Shearmur, R. (2019). Diverse diversities—Open innovation in small towns and rural areas. *Growth and Change,**50*(2), 492–514. 10.1111/grow.12291

[CR79] Mitra, M., de Vos, M. G., Cortinovis, N., & Ometto, D. (2024). Generative AI for Research Data Processing: Lessons Learnt From Three Use Cases. *2024 IEEE 20th International Conference on E-Science (e-Science)*, 1–10. 10.1109/e-Science62913.2024.10678704

[CR80] Mollick, E. (2014). The dynamics of crowdfunding: An exploratory study. *Journal of Business Venturing,**29*(1), 1–16. 10.1016/j.jbusvent.2013.06.005

[CR81] Mollick, E., & Nanda, R. (2016). Wisdom or madness? Comparing crowds with expert evaluation in funding the arts. *Management Science,**62*(6), 1533–1553.

[CR82] Mollick, E., & Robb, A. (2016). Democratizing innovation and capital access: The role of crowdfunding. *California Management Review,**58*(2), 72–87. 10.1525/cmr.2016.58.2.72

[CR83] Mollick, E., & Kuppuswamy, V. (2016). Crowdfunding: Evidence on the Democratization of Start-up Funding. In *Revolutionizing Innovation: Users, Communities, and Open Innovation* (pp. 537–559). MIT Press. https://direct.mit.edu/books/edited-volume/4075/chapter/169013/Crowdfunding-Evidence-on-the-Democratization-of

[CR84] Moyes, D., Ferri, P., Henderson, F., & Whittam, G. (2015). The stairway to heaven? The effective use of social capital in new venture creation for a rural business. *Journal of Rural Studies,**39*, 11–21. 10.1016/j.jrurstud.2015.02.004

[CR85] Müller, S., & Korsgaard, S. (2018). Resources and bridging: The role of spatial context in rural entrepreneurship. *Entrepreneurship & Regional Development,**30*(1–2), 224–255. 10.1080/08985626.2017.1402092

[CR86] Muñoz, P., & Kimmitt, J. (2019a). Rural entrepreneurship in place: An integrated framework. *Entrepreneurship & Regional Development,**31*(9–10), 842–873. 10.1080/08985626.2019.1609593

[CR87] Muñoz, P., & Kimmitt, J. (2019b). Social mission as competitive advantage: A configurational analysis of the strategic conditions of social entrepreneurship. *Journal of Business Research,**101*, 854–861. 10.1016/j.jbusres.2018.11.044

[CR88] Naldi, L., Nilsson, P., Westlund, H., & Wixe, S. (2021). Amenities and new firm formation in rural areas. *Journal of Rural Studies,**85*, 32–42. 10.1016/j.jrurstud.2021.05.023

[CR89] Nambisan, S., Wright, M., & Feldman, M. (2019). The digital transformation of innovation and entrepreneurship: Progress, challenges and key themes. *Research Policy,**48*(8), Article 103773. 10.1016/j.respol.2019.03.018

[CR90] North, D. (2005). *Understanding the Process of Economic Change*. Princeton University Press.

[CR91] Paschen, J. (2017). Choose wisely: Crowdfunding through the stages of the startup life cycle. *Business Horizons,**60*(2), 179–188. 10.1016/j.bushor.2016.11.003

[CR92] Pominova, M., Gabe, T., & Crawley, A. (2022). The stability of location quotients. *Review of Regional Studies*, *52*(3). 10.52324/001c.66197

[CR93] Putnam, R. (2001). *Bowling alone*. Simon & Schuster.

[CR94] Qiao, Y., Cortinovis, N., & Morrison, A. (2024). MNE spillovers and local export dynamics in China: The role of relatedness and forward–backward linkages. *Eurasian Business Review*. 10.1007/s40821-024-00273-8

[CR95] Ratcliffe, M., Burd, C., Holder, K., & Fields, A. (2016). Defining Rural at the U.S. Census Bureau. *ACSGEO-1*.

[CR96] Ring, J. K., Peredo, A. M., & Chrisman, J. J. (2010). Business networks and economic development in rural communities in the United States. *Entrepreneurship Theory and Practice,**34*(1), 171–195. 10.1111/j.1540-6520.2009.00307.x

[CR97] Romero-Castro, N., López-Cabarcos, M. A., & Piñeiro-Chousa, J. (2023). Finance, technology, and values: A configurational approach to the analysis of rural entrepreneurship. *Technological Forecasting and Social Change,**190*, Article 122444. 10.1016/j.techfore.2023.122444

[CR98] Rupasingha, A., Goetz, S. J., & Freshwater, D. (2006). The production of social capital in US counties. *The Journal of Socio-Economics,**35*(1), 83–101. 10.1016/j.socec.2005.11.001

[CR99] Schrijvers, M., Stam, E., & Bosma, N. (2024). Figuring it out: Configurations of high-performing entrepreneurial ecosystems in Europe. *Regional Studies,**58*(5), 1096–1110. 10.1080/00343404.2023.2226727

[CR100] Schwartz, A. A. (2012). Rural crowdfunding. *UC Davis Business Law Journal,**13*(2), 283–294.

[CR101] Shearmur, R. (2012). Are cities the font of innovation? A critical review of the literature on cities and innovation. *Cities,**29*, S9–S18. 10.1016/j.cities.2012.06.008

[CR102] Smallbone, D., North, D., Roper, S., & Vickers, I. (2003). Innovation and the use of technology in manufacturing plants and SMEs: An interregional comparison. *Environment and Planning c, Government and Policy,**21*(1), 37–52. 10.1068/c0218

[CR103] Smith, C., Smith, J. B., & Shaw, E. (2017). Embracing digital networks: Entrepreneurs’ social capital online. *Journal of Business Venturing,**32*(1), 18–34. 10.1016/j.jbusvent.2016.10.003

[CR104] Sorenson, O., Assenova, V., Li, G.-C., Boada, J., & Fleming, L. (2016). Expand innovation finance via crowdfunding. *Science,**354*(6319), 1526–1528. 10.1126/science.aaf6989 28008025 10.1126/science.aaf6989

[CR105] Spigel, B. (2017). The relational organization of entrepreneurial ecosystems. *Entrepreneurship Theory and Practice,**41*(1), 49–72. 10.1111/etap.12167

[CR106] Spigel, B., & Harrison, R. (2018). Toward a process theory of entrepreneurial ecosystems. *Strategic Entrepreneurship Journal,**12*(1), 151–168. 10.1002/sej.1268

[CR107] Stam, E., & Van De Ven, A. (2021). Entrepreneurial ecosystem elements. *Small Business Economics,**56*(2), 809–832. 10.1007/s11187-019-00270-6

[CR108] Tian, Z. (2013). Measuring agglomeration using the standardized location quotient with a bootstrap method. *The Journal of Regional Analysis & Policy,**43*(2), 186–197.

[CR109] Ughetto, E., Cowling, M., & Lee, N. (2019). Regional and spatial issues in the financing of small and medium-sized enterprises and new ventures. *Regional Studies,**53*(5), 617–619. 10.1080/00343404.2019.1601174

[CR110] van Rijnsoever, F. J. (2020). Meeting, mating, and intermediating: How incubators can overcome weak network problems in entrepreneurial ecosystems. *Research Policy,**49*(1), Article 103884. 10.1016/j.respol.2019.103884

[CR111] Virkkala, S. (2007). Innovation and networking in peripheral areas—A case study of emergence and change in rural manufacturing. *European Planning Studies,**15*(4), 511–529. 10.1080/09654310601133948

[CR112] Wojan, T. R., & Nichols, B. (2018). Design, innovation, and rural creative places: Are the arts the cherry on top, or the secret sauce? *PLoS ONE,**13*(2), Article e0192962. 10.1371/journal.pone.0192962 29489884 10.1371/journal.pone.0192962 PMC5831055

[CR113] Yaşlak, B., Akgün, A. A., & Baycan, T. (2023). Social networks of online rural entrepreneurs: The case of Turkey. *The Annals of Regional Science,**70*(3), 705–721. 10.1007/s00168-020-01034-x

[CR114] Yu, S., Johnson, S., Lai, C., Cricelli, A., & Fleming, L. (2017). Crowdfunding and regional entrepreneurial investment: An application of the CrowdBerkeley database. *Research Policy,**46*(10), 1723–1737. 10.1016/j.respol.2017.07.008

